# Diastereoisomer-Specific Biotransformation of Hexabromocyclododecanes by a Mixed Culture Containing *Dehalococcoides mccartyi* Strain 195

**DOI:** 10.3389/fmicb.2018.01713

**Published:** 2018-07-30

**Authors:** Yin Zhong, Heli Wang, Zhiqiang Yu, Xinhua Geng, Chengyu Chen, Dan Li, Xifen Zhu, Huajun Zhen, Weilin Huang, Donna E. Fennell, Lily Y. Young, Ping’an Peng

**Affiliations:** ^1^State Key Laboratory of Organic Geochemistry, Guangzhou Institute of Geochemistry, Chinese Academy of Sciences, Guangzhou, China; ^2^University of Chinese Academy of Sciences, Beijing, China; ^3^School of Chemistry and Chemical Engineering, Guangzhou University, Guangzhou, China; ^4^College of Natural Resources and Environment, South China Agricultural University, Guangzhou, China; ^5^Department of Environmental Sciences, Rutgers, The State University of New Jersey, New Brunswick, NJ, United States

**Keywords:** anaerobic degradation, debromination, stereoisomer, enantiomers, HBCD

## Abstract

Hexabromocyclododecane (HBCD) stereoisomers may exhibit substantial differences in physicochemical, biological, and toxicological properties. However, there remains a lack of knowledge about stereoisomer-specific toxicity, metabolism, and environmental fate of HBCD. In this study, the biotransformation of (±)α-, (±)β-, and (±)γ-HBCD contained in technical HBCD by a mixed culture containing the organohalide-respiring bacterium *Dehalococcoides mccartyi* strain 195 was investigated. Results showed that the mixed culture was able to efficiently biotransform the technical HBCD mixture, with 75% of the initial HBCD (∼12 μM) in the growth medium being removed within 42 days. Based on the metabolites analysis, HBCD might be sequentially debrominated via dibromo elimination reaction to form tetrabromocyclododecene, dibromocyclododecadiene, and 1,5,9-cyclododecatriene. The biotransformation of the technical HBCD was likely diastereoisomer-specific. The transformation rates of α-, β-, and γ-HBCD were in the following order: α-HBCD > β-HBCD > γ-HBCD. The enantiomer fractions of (±)α-, (±)β-, and (±)γ-HBCD were maintained at about 0.5 during the 28 days of incubation, indicating a lack of enantioselective biotransformation of these diastereoisomers. Additionally, the amendment of another halogenated substrate tetrachloroethene (PCE), which supports the growth of strain 195, had a negligible impact on the transformation patterns of HBCD diastereoisomers and enantiomers. This study provided new insights into the stereoisomer-specific transformation patterns of HBCD by anaerobic microbes and has important implications for microbial remediation of anoxic environments contaminated by HBCD using the mixed culture containing *Dehalococcoides*.

## Introduction

Hexabromocyclododecane (HBCD) is a widely used cyclic aliphatic brominated flame retardant found in polymers, textiles, electronic, and electric products. Due to its persistence in the environment and the associated environmental and human health risks (Marvin et al., 2011; Koch et al., 2015), HBCD has been included in the Annex A as a persistent organic pollutant (POP) by the Stockholm Convention (United Nations Environment Programme [UNEP], 2013). Currently, the production and use of HBCD have been banned in many countries (United Nations Environment Programme [UNEP], 2013). It is necessary to better understand the fate of HBCD released to the environment and to develop effective methods to remediate sites contaminated by HBCD.

**FIGURE 1:**
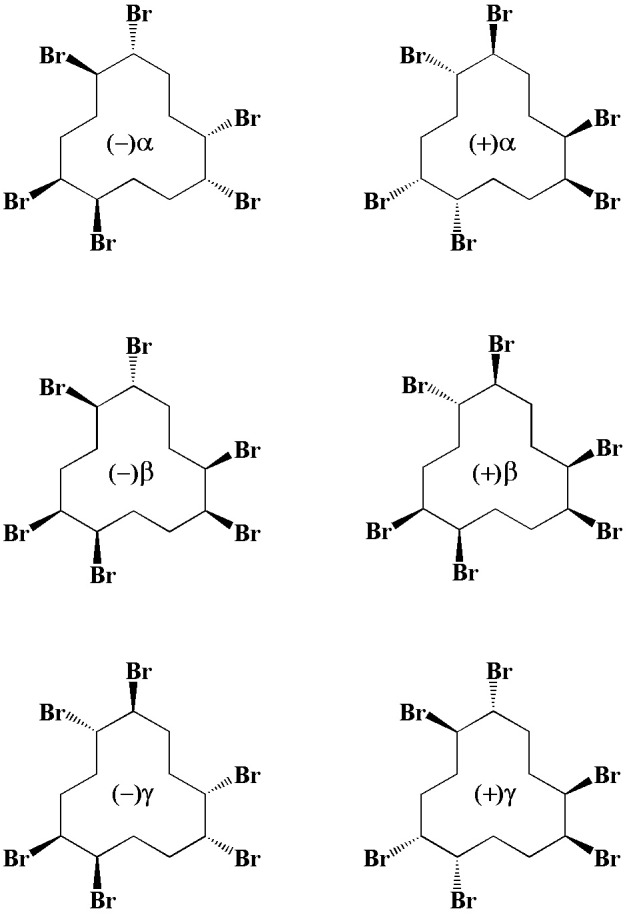
Structure of (±)α-, (±)β-, and (±)γ-HBCD.

Technical-grade HBCD is synthesized by bromination of 1,5,9-cyclododecatriene (Heeb et al., 2005), which theoretically leads to a mixture of three major diastereomeric pairs of enantiomers, i.e., (±)α-, (±)β-, and (±)γ-HBCD. The structures of (±)α-, (±)β-, and (±)γ-HBCD are shown in **Figure 1**. Different physiochemical properties (e.g., polarity, water solubility, and dipole moment) of these HBCD stereoisomers may lead to substantial differences in their toxicity, metabolism, and environmental fate (Hakk et al., 2012). Indeed, there is increasing evidence for the diastereoisomer- and/or enantiomer-specific distribution and accumulation in various environmental media (Yu et al., 2008; Haukås et al., 2009; Gao et al., 2011; Zhang et al., 2011; Wu et al., 2014), biota (Janák et al., 2005; Zheng et al., 2017), and even in human body (Shi et al., 2009).

Due to its highly hydrophobic nature (log *K*_ow_ = 5.6), HBCD released to aquatic environments tends to partition to and be accumulated in anoxic sediments (Davis et al., 2005; Law et al., 2014). Anaerobic transformation and degradation is considered as a key pathway of natural attenuation of HBCD under anoxic conditions (Davis et al., 2005, 2006; Gerecke et al., 2006; Stiborova et al., 2015). Experiments with river sediments have shown faster rates of HBCD transformation in anaerobic conditions than in aerobic conditions, with β-HBCD transformation being faster than α- and γ-HBCD (Davis et al., 2005, 2006). However, very little is known about the transformability of HBCD by pure or mixed cultures of anaerobic microbes. Until now, only two HBCD-degrading bacteria have been isolated (Peng et al., 2015) and no mixed cultures containing anaerobic microbes have been demonstrated to be able to debrominate HBCD.

Organohalide-respiring bacteria (OHRB) are key players in natural attenuation of halogenated organic compounds in anoxic environments (Löffler et al., 2013; Wang et al., 2014; Adrian and Löffler, 2016). *Dehalococcoides* is the best-studied OHRB that can degrade halogenated POPs (Adrian and Löffler, 2016). *Dehalococcoides mccartyi* strain 195 (formerly *Dehalococcoides ethenogenes* strain 195) is the first bacterium known to completely dechlorinate tetrachloroethene (PCE) to ethene as the sole electron acceptor for growth (Maymó-Gatell et al., 1997; Löffler et al., 2013). It can cometabolize a broad variety of recalcitrant POPs in mixed cultures or in the presence of other electron acceptors, such as polychlorinated biphenyls (PCBs), polychlorinated dibenzo-*p*-dioxins (PCDD/Fs) and polybrominated diphenyl ethers (PBDEs) (Fennell et al., 2004; He et al., 2006; Liu and Fennell, 2008; Zhen et al., 2014). *D. mccartyi* strain CBDB1 is capable of debrominating many brominated compounds, such as tetra- and penta-brominated diphenyl ether, tetrabromobisphenol A, bromophenol blue, and hexabromobenzene (Lee et al., 2011; Yang et al., 2015). Our previous study showed that mixed cultures containing *D. mccartyi* strain 195 dehalogented halogenated compounds more efficiently than the pure culture (Fennell et al., 2004). Further, the organisms have been utilized successfully in the engineered remediation of natural environments contaminated by halogenated compounds (Schaefer and Steffan, 2016).

Therefore, this study was designed to examine the mixed culture containing *D. mccartyi* strain 195 for their ability to biotransform HBCD as the sole halogenated substrate or in the presence of PCE. Special attention was given to the change in the shift pattern transformation characteristics of diastereoisomers and enantiomers of HBCD. The results obtained here will give new insight into the biotransformation patterns of HBCD stereoisomers by OHRB under anaerobic conditions and facilitate the practical application of OHRB during bioremediation of HBCD-contaminated anoxic environments.

## Materials and Methods

### Chemicals and Culture Preparation

The technical HBCD mixture was purchased from Tokyo Chemical Industry (Tokyo, Japan). Hexabromobenzene (97%) was purchased from Alfa Aesar (Haverhill, MA, United States). Hexane (Merck, Darmstadt, Germany), isooctane (Alfa Aesar, Haverhill, MA, United States), and toluene (JT Baker, Phillipsburg, NJ, United States) were used as received.

The mixed culture containing *D. mccartyi* strain 195 was cultured in the presence of butyric acid and tetrachloroethene (PCE) as the electron donor and acceptor under anaerobic condition, respectively (Zhen et al., 2014). The quantitative polymerase chain reaction (qPCR) analysis and high throughput sequencing analysis showed that *D. mccartyi* strain 195 still was the only dehalogenating bacteria detected in the mixed culture, which was consistent with the result of our previous study (Fennell et al., 2004; Krumins et al., 2009; Zhen et al., 2014).

The composition of growth medium of this mixed culture was described previously by Fennell (1998). Briefly, the medium consisted of NH_4_Cl (0.2 g L^-1^), K_2_HPO_4_ (0.1 g L^-1^), KH_2_PO_4_ (0.055 g L^-1^), MgCl_2_⋅6H_2_O (0.2 g L^-1^), FeCl_2_⋅4H_2_O (0.1 g L^-1^), Na_2_S⋅9H_2_O (0.5 g L^-1^), NaHCO_3_ (6 g L^-1^), resazurin (0.001 g L^-1^), vitamin stock solutions and trace metal solution.

### Biotransformation Experiment

A batch experiment was conducted for quantifying the transformability of HBCD by the mixed culture. Serum bottles (60 ml) sealed with a Teflon-coated gray butyl rubber stopper and crimped with an aluminum crimp cap were used as the batch reactor. Firstly, HBCD stock solution (1 mL) was added to each bottle containing 1 g of dry and sterile silica powder via a sterile glass syringe. After the solvent was evaporated under sterile and anaerobic conditions, the bottles were purged for 15 min with anoxic and sterile gas mixture (70% N_2_/30% CO_2_). The mixed culture (50 mL), with the density of *D. mccartyi* strain 195 at approximately 2 × 10^8^ cells mL^-1^, was transferred into each bottle under sterile and anaerobic conditions. The initial concentration of HBCD in the medium was ∼12 μM. Four sets of triplicate bottles were set up at 30°C. One set of bottles was amended with active cells and HBCD as the sole halogenated compound. The second set of bottles was amended with active cells, HBCD and PCE for testing the effects of PCE as an alternative electron acceptor (also a growth substrate) on the biotransformation of HBCD. The third set of bottles was amended with active cells and PCE as the sole halogenated compound. The fourth set of bottles were control reactors containing HBCD and cells killed by autoclaving for 30 min on each of three consecutive days. All treatments were conducted in triplicate. In addition, 100 μL of neat butyric acid and 50 μL of fermented yeast extract solution (50 g L^-1^) were added into all bottles as carbon source and nutrient source every 2 weeks, respectively (Zhen et al., 2014). All bottles were shaken at 250 rpm on a shaker placed in the darkroom at 30°C. At predetermined time intervals, an aliquot of 1 mL of the cultures were sampled from each bottle via sterile and anoxic syringe and was freeze-dried for the analysis of residual concentration of HBCD, HBCD diastereoisomers, and enantiomers.

In order to identify the degradation products of HBCD, a separate experiment was performed using higher initial concentration of HBCD (∼38 μM) so that sufficient mass of the products was obtained. The other experimental conditions were kept the same as those of above batch experiments. At predetermined time, an aliquot of 1 mL of the cultures were sampled from each bottle via sterile and anoxic syringe and was freeze-dried for the analysis of HBCD products.

### Analytical Methods

An Agilent 6890N network GC system equipped with Agilent 5973N network mass selective detector was employed for the determination of residual HBCD and potential low debrominated products. An Agilent 1100 series HPLC system with a API 4000 triple quadrupole mass spectrometer (LC-MS/MS) with a TurboIonSpray ionization interface was employed for the determination of HBCD diastereoisomers and enantiomers.

For quantifying concentrations of residual HBCD, the freeze-dried samples were spiked with 20 μL of 450 μM hexabromobenzene as the recovery surrogate and ultrasonically extracted twice with 1 mL of isooctane/hexane mixtures (9:1, v/v) for 15 min. Then, the supernatants were combined, concentrated and analyzed by GC-MS equipped with an on-column injector and a DB-5 capillary GC column (15 m length, 0.25 mm id and 0.1 μm film thickness) operating in negative chemical ionization (NCI) mode. The ions with m/z 79, 81, 561, and 563 were selectively monitored. A cold on-column injector was operated in track-oven mode. The oven operation temperature was set from 60°C for 1 min, increased to 260°C at 10°C min^-1^, and held at 260°C for 5 min, and then increased to 320°C at 20°C min^-1^. The ion source and quadrupole temperatures were set at 150 and 200°C, respectively. Helium was used as the carrier gas at a flow rate of 1.2 mL min^-1^, and methane was used as the moderating gas. Quality assurance was performed by the analyses of spiked blanks. The recoveries of HBCD and hexabromobenzene in spiked blanks ranged from 96 to 108% and 98 to 113%, respectively. Limits of detection (LOD) and limits of quantification (LOQ) were defined a signal-to-noise ratio of 3 and 10, respectively. LOD and LOQ of HBCD were 0.03 and 0.11 ng, respectively.

For identifying potential debrominated products, triplicate samples were combined, concentrated, and analyzed by GC-MS equipped with a Rtx-5 ms fused silica capillary column (30 m length, 0.25 mm id, and 0.25 μm film thickness) operating in electron ionization (EI) mode. The GC column temperature was programmed to maintain at 80°C for 1 min, ramped at 10°C min^-1^, to 300°C, and held at 300°C for 10 min. The temperature of injector was set to 250°C. The carrier gas was helium at a flow rate of 1 mL min^-1^. Both ion source and GC-MS-EI interface temperature were held isothermally at 250°C. The mass spectrometer was run in electron impact ionization mode (70 eV) and was scanned from 45 to 700 amu.

Three major HBCD diastereoisomers (α-, β-, and γ-HBCD) and their enantiomers were analyzed using a LC-MS/MS according to the method described by Yu et al. (2008). The enantiomeric composition was expressed as enantiomeric fraction (EF). In order to avoid the matrix effect, ^13^C-labeled α-, β-, and γ-HBCD were added as internal standards for both quantification and correction of EF values. The corrected EFs were calculated by the following equation:

$$\text{EF}=\frac{A_{+}/A_{+\text{c}13}}{A_{+}/A_{+\text{c}13}+A_{-}/A_{-\text{c}13}}$$

where *A*_+_ and *A*_-_ represent the peak areas of (+)-enantiomer and (-)-enantiomer, respectively, while *A*_+C13_ and *A*_-C13_ represent the peak areas of (+)-enantiomer and (-)-enantiomer labeled by ^13^C, respectively.

## Results and Discussion

**FIGURE 2:**
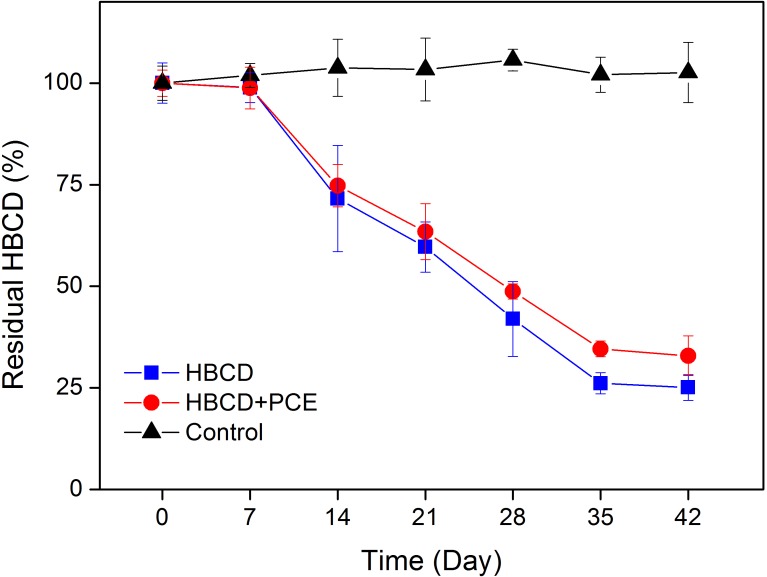
Anaerobic transformation of the technical HBCD by the mixed culture in the presence or absence of PCE. The initial concentration of HBCD coating on the silicate in the growth medium was 12 μM.

The mixed culture containing *D. mccartyi* strain 195 have demonstrated the ability of dehalogenating recalcitrant POPs like PCBs and PCDD/Fs (Liu and Fennell, 2008; Zhen et al., 2014), which might also have the potential to transform HBCD. As shown in **Figure 2**, when technical HBCD was added as the sole halogenated amendment to the mixed culture, loss of the compounds was observed, supporting our hypothesis. After a lag period of approximately 7 days, the mixed culture exhibited high activity for the biotransformation of the technical HBCD. After 42 days of incubation, 75% of the initial HBCD (12 μM, equal to 7704 μg L^-1^) in the growth medium of the live mixed culture disappeared. There was no significant degradation between 35 and 42 days. It was likely because the residual concentration of HBCD as the electron acceptor was too low to sustain the growth of dehalogenating bacteria, therefore, the degradation rate did not increase. Indeed, a similar phenomenon was reported in a previous study (Cupples et al., 2004). No apparent HBCD removal was observed in the killed control throughout the experiment period.

**FIGURE 3:**
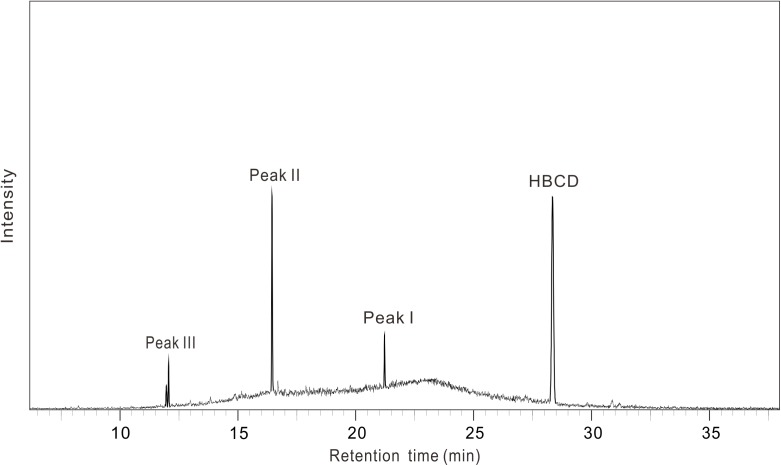
Formation of degradation products of HBCD by the mixed culture.

**FIGURE 4:**
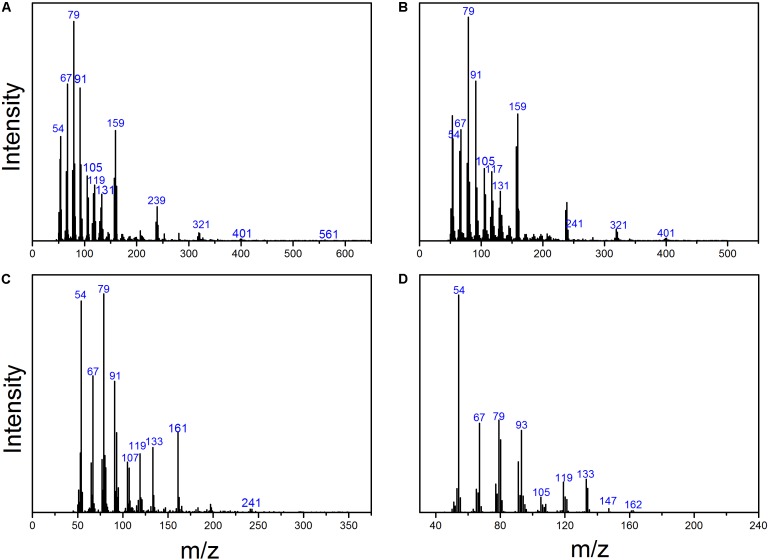
Mass spectrum of HBCD and its debromination products. **(A)** HBCD; **(B)** Peak I, tetrabromocyclododecene; **(C)** Peak II, dibromocyclododecadiene; and **(D)** Peak III, 1,5,9-cyclododecatriene.

**FIGURE 5:**
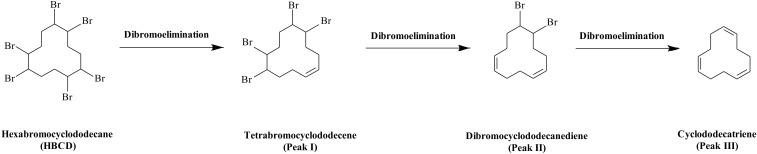
The proposed debromination pathway of HBCD by the mixed culture.

As shown in **Figure 3**, there were three metabolites produced during anaerobic degradation of HBCD, i.e., Peaks I, II, and III. As shown in **Figure 4**, Peaks I and II were tentatively identified as tetrabromocyclododecene and dibromocyclododecadiene, respectively, by comparison with mass spectral of debromination products of HBCD reported in previous studies (Li et al., 2016, 2017). Peak III was identified by 1,5,9-cyclododecatriene by comparison with the respective retention time and mass spectrum of external standards. As shown in the proposed debromination pathway (**Figure 5**), HBCD was sequentially debrominated by anaerobic bacteria via dibromo elimination reaction to form tetrabromocyclododecene, dibromocyclododecadiene, and 1,5,9-cyclododecatriene. Similar debromination products and pathway have been reported in anaerobic degradation of HBCD by pure culture of *Achromobacter* sp. as well as in digester sludge and freshwater aquatic sediments (Davis et al., 2006; Peng et al., 2015).

Our observations are consistent with a prior report which showed *Achromobacter* sp. HBCD-1 could anaerobically transform 35.1% of 5000 μg L^-1^ HBCD in its growth medium within 8 days (Peng et al., 2015). However, it is difficult to make a detailed comparison of our observations with other studies of transformation of HBCD by OHRB due to limited published data. Indeed, it is interesting to show that *Dehalococcoides* organisms utilized HBCD relatively rapidly compared to PBDEs (He et al., 2006). As shown by He et al. (2006), pure and enriched culture containing *D. mccartyi* strain 195 exhibited detectable debromination activity of octa-BDEs within 6 and 3 months, respectively. In previous studies, when more hydrophobic POPs were added as the sole electron acceptors (Fennell et al., 2004; He et al., 2006; Liu and Fennell, 2008; Zhen et al., 2014), the rates of dechlorination were slower. For instance, it was shown that less than 20% of the initial 1,2,3,4,7,8-hexachlorodibenzofuran in growth medium was dechlorinated to less chlorinated daughter products by a mixed culture containing *D. mccartyi* strain 195 after 195 days of incubation (Liu and Fennell, 2008). Nonetheless, our results mentioned above, in combination with the fact that *D. mccartyi* strain 195 has 17 putative reductive dehalogenase genes (Seshadri et al., 2005), warrant further study on the potential activities of *Dehalococcoides* organisms for the degradation of other POPs and/or halogenated contaminants.

**FIGURE 6:**
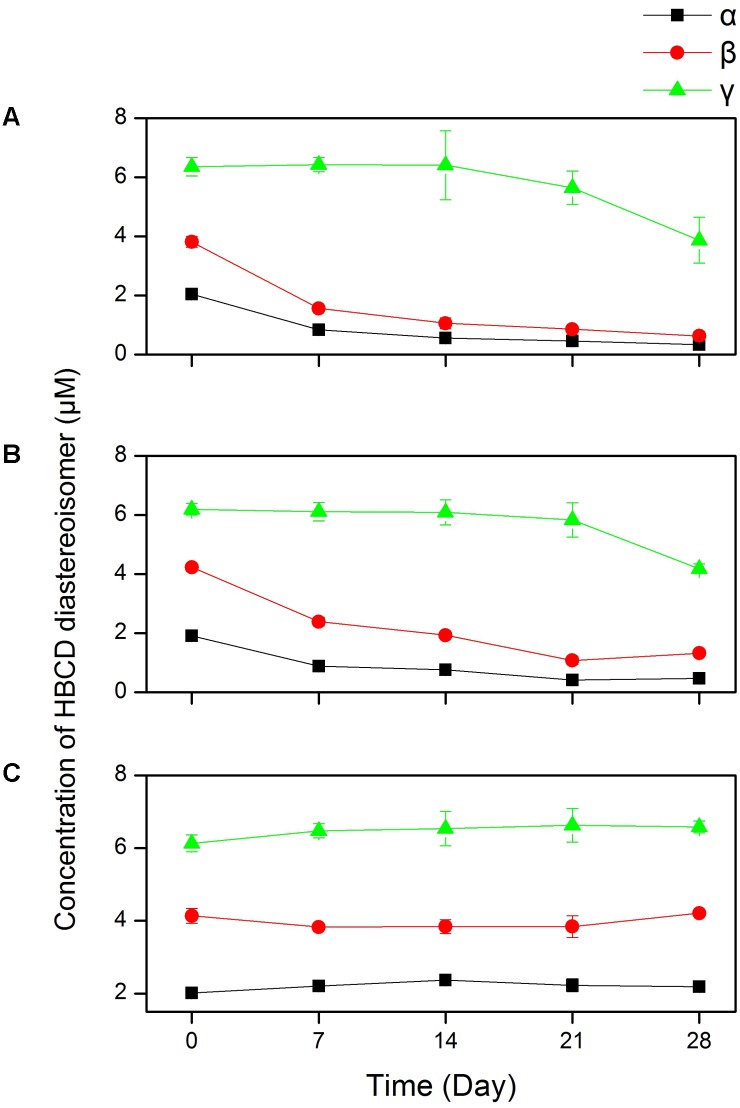
The residual concentration of three HBCD diastereoisomers during the degradation of HBCD by the mixed culture when HBCD was amended as the sole halogenated substrate **(A)**, HBCD co-existed with PCE **(B)**, and in control with killed cells **(C)** in the growth medium.

Note that the biotransformation of the technical HBCD by the mixed culture containing *D. mccartyi* strain 195 was likely diastereoisomer-specific (**Figure 6**). After 28 days of incubation, the remaining percentages of α-HBCD, β-HBCD, and γ-HBCD were 16, 26, and 60%, respectively (**Figure 6A**). It suggested that the transformation rate of three HBCD diastereoisomers followed the order of α-HBCD > β-HBCD > γ-HBCD. The pattern of transformation rates of α-, β-, and γ-HBCD by the mixed culture used in this study (**Figure 6A**) was consistent with that of the three diastereoisomers by the anaerobic bacterium *Achromobacter* sp. HBCD-1 (Peng et al., 2015). In contrast, it was reported that the anaerobic biotransformation of α-HBCD in sewage sludge (Gerecke et al., 2006) and river sediments (Davis et al., 2006) was slower than that of β-HBCD and γ-HBCD. These different outcomes could be caused by a number of environmental factors. It is highly likely that other microbes, rather than *Dehalococcoides* or *Achromobacter* organisms, may be able to debrominate HBCD via different mechanisms in the sewage sludge and river sediments. It is also apparent that the initial concentrations of HBCD diastereoisomers in the technical HBCD mixtures used are likely very different from those present in the environments, causing varied rates of biotransformation of different diastereoisomers (Davis et al., 2006). Interestingly, α-HBCD with higher hydrophobicity was adsorbed more strongly on sediment particles in the environments (Marvin et al., 2011), rendering lower bioavailability of α-HBCD and hence retarded degradation rate in the environments. The diastereoisomer-specific degradation of HBCD in the presence of PCE by the mixed culture is shown in **Figure 6B** and discussed below in detail. No diastereoisomer-specific transformation was observed in control (**Figure 6C**).

**FIGURE 7:**
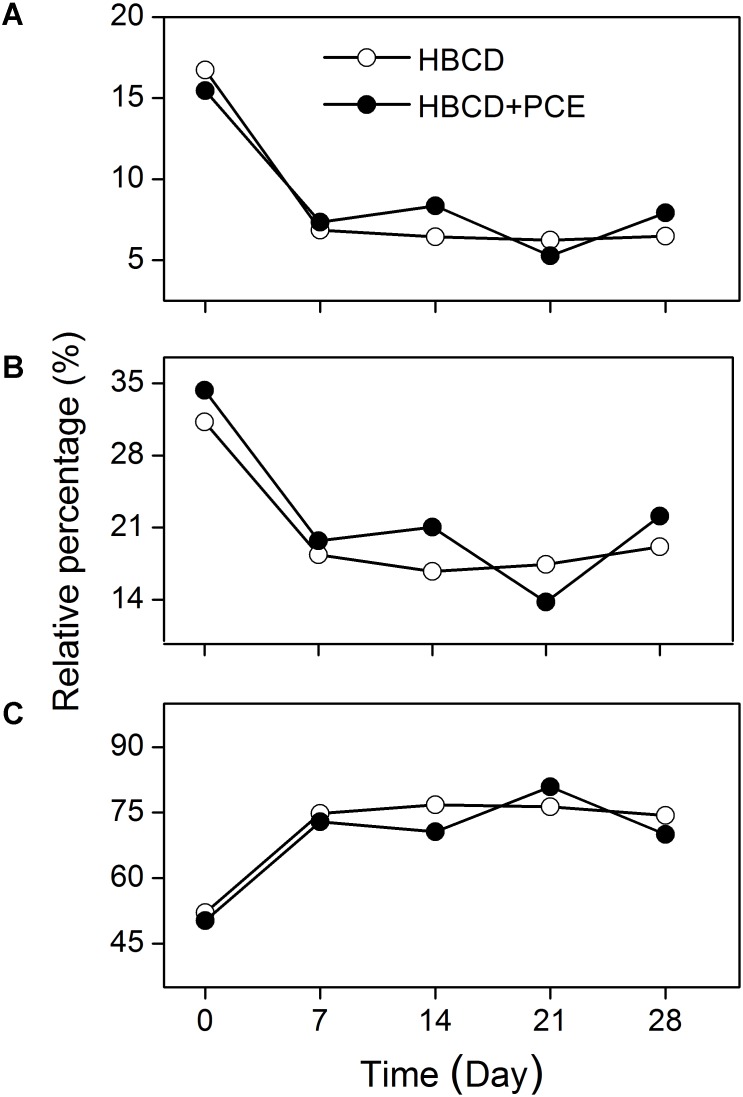
The dynamics of the proportions of α-HBCD **(A)**, β-HBCD **(B)**, and γ-HBCD **(C)** to the total HBCD throughout the transformation experiment, where HBCD was degraded as the sole halogenated substrate or in the presence of PCE by the mixed culture.

For better understanding the effect of diastereoisomer-specific biotransformation on the change in stereoisomeric composition of HBCD, the dynamics of the proportions of individual diastereoisomers to the total HBCD throughout the debromination experiment should be considered. The proportion of γ-HBCD was the largest among the diastereoisomers and increased with time (**Figure 7C**), whilst those of all the other diastereoisomers tended to decrease with time (**Figures 7A,B**). In general, these results presented a plausible explanation for the observation that γ-HBCD was the most dominant diastereoisomer in many anoxic sediments (Harrad et al., 2009; Gao et al., 2011; Meng et al., 2011; Zhang et al., 2011; Klosterhaus et al., 2012; Wu et al., 2014). However, the increased proportion of γ-HBCD observed in this study was inconsistent with the phenomenon that the proportion of γ-HBCD in some river sediments was much lower than those of technical HBCD mixtures (Wu et al., 2014). This discrepancy could be explained by a scenario that abiotic transformation played an important role in the distribution and accumulation of HBCD diastereoisomers in the sediments, further reflecting the complexity of factors influencing the environmental fates of HBCD diastereoisomers.

Enantiomeric selectivity of HBCD played key role in the toxicity and fate of HBCD stereoisomers in the environment. There is strong evidence that the environmental fates of HBCD are enantioselective (Zhang et al., 2011; Zheng et al., 2017), while enantiomeric analysis has received relatively little attention in the literature on anaerobic degradation of HBCD. In this study, the occurrences of three pairs of enantiomers in the technical HBCD mixture, i.e., (±)α-, (±)β-, and (±)γ-HBCD, were confirmed. Further, EFs of the three HBCD diastereoisomers were calculated to determine whether enantiomeric selectivity was involved in the HBCD transformation by the mixed culture. As shown in **Table 1**, the ranges of EFs of α-, β-, and γ-HBCD throughout the transformation experiment were 0.49–0.52, 0.50–0.51, and 0.50–0.51, respectively. That is, the EFs of the three diastereoisomers were close to 0.5, suggesting that the anaerobic transformation of HBCD was not an enantioselective process. Similarly, no enantioselective pattern was observed in the anaerobic degradation of HBCD in sewage sludge (Gerecke et al., 2006). However, the racemic α-, β-, and γ-HBCD could undergo enantioselective degradation under aerobic conditions (Heeb et al., 2014, 2017). For instance, an aerobic HBCD-degrading bacterium (*Sphingobium chinhatense* IP26) could transform (+)α-, (-)β-, and (-)γ-HBCD at a faster rate than those of their enantiomers (Heeb et al., 2017). Dehalogenase LinB from *Sphingobium indicum* B90A transformed (-)α-, (+)β-, and (+)γ-HBCD at faster rates than those of their enantiomers (Heeb et al., 2012), whilst dehalogenase LinA could transform only (-)β-HBCD substantially (Heeb et al., 2014). In addition, enantioselective transformation and enrichment of HBCD were even observed in animals (Janák et al., 2005; Du et al., 2012). These findings indicate that mechanisms of anaerobic biotransformation of HBCD enantiomers are considerably different from those of aerobic biodegradation.

**Table 1:** Enantiomer fractions (EFs) of α-, β-, and γ-HBCD during the transformation of the technical HBCD mixture by the mixed culture containing *D. mccartyi* strain 195.

Treatment	Time (Day)	EF_α_	EF_β_	EF_γ_
Control	0	0.51	0.49	0.51
	7	0.52	0.51	0.51
	14	0.52	0.51	0.51
	21	0.51	0.51	0.50
	28	0.53	0.50	0.50
HBCD	0	0.52	0.50	0.50
	7	0.49	0.50	0.50
	14	0.52	0.50	0.51
	21	0.52	0.50	0.51
	28	0.52	0.51	0.51
PCE+HBCD	0	0.52	0.50	0.50
	7	0.51	0.50	0.50
	14	0.52	0.51	0.50
	21	0.52	0.50	0.51
	28	0.49	0.51	0.50

**FIGURE 8:**
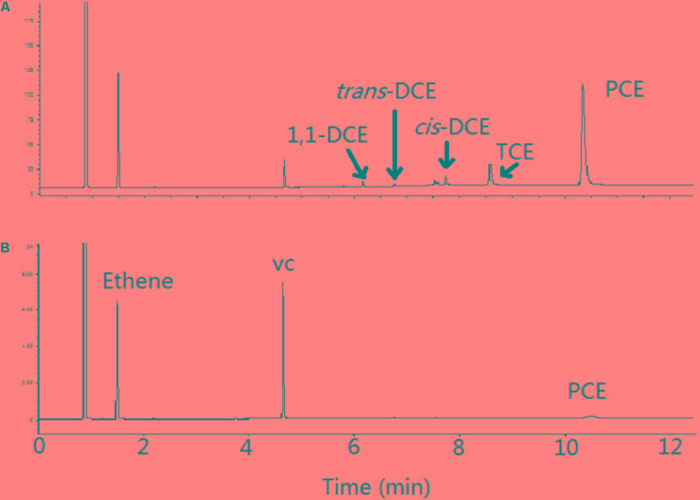
Degradation of PCE and its intermediates by the mixed culture in presence of HBCD at day 7 **(A)** and day 28 **(B)**.

Co-amendment of halogenated growth substrate (haloprimer) has been shown to stimulate the dehalogenating activity of *D. mccartyi* strain 195 for the debromination of recalcitrant halogenated compounds such as 1,2,3,4,7,8-hexachlorodibenzofuran (Ahn et al., 2008; Liu and Fennell, 2008). However, in this study, the biotransformation rate of the technical HBCD by the mixed culture was not enhanced when PCE was amended as an additional halogenated substrate (**Figure 2**). In addition, the presence of PCE had negligible effects on the transformation patterns of individual diastereoisomers and enantiomers (**Figures 6**, **7**, and **Table 1**). On the other hand, the degradation of PCE by the mixed culture and dechlorinated products (e.g., TCE, DCE, and vinyl chloride) was hardly influenced by the presence of HBCD after 28-day of incubation (**Figure 8**). These results indicate that the dehalogenases responsible for the debromination of HBCD by the mixed culture might be different from those of PCE. Another important implication of these results is that the addition of co-substrate PCE to stimulate the transformation of HBCD might not be useful when the mixed culture containing *D. mccartyi* strain 195 is applied into the remediation of anoxic environments contaminated by HBCD.

## Conclusion

This study demonstrated that the mixed culture containing *D. mccartyi* strain 195 can relatively rapidly transform the technical HBCD mixture when added as the sole halogenated substrate and that the loss/transformation is stereoisomer-specific. It suggested that the transformation rate of three HBCD diastereoisomers followed the order of α-HBCD > β-HBCD > γ-HBCD. The pathway of HBCD degradation has been proposed. The results also showed that addition of PCE as a co-substrate had negligible effect on the removal of both PCE and HBCD by the mixed culture. This may indicate that *D. mccartyi* strain 195 utilizes different enzymes for the biotransformation of HBCD and PCE. The results not only provide insight to the environmental fate of HBCD stereoisomers and mechanistic understanding of biotransformation of HBCD, but also facilitate the practical guidance for design of bioremediation schemes by using halogen-respirators to treat environments contaminated with HBCD.

## Author Contributions

PP, LY, and WH designed the project. YZ, ZY, HW, XG, CC, DL, XZ, and HZ performed the experiments and data analyses. DF provided the culture. YZ wrote the manuscript. PP, LY, DF, ZY, and WH reviewed the manuscript. All authors have read and approved the manuscript.

## Conflict of Interest Statement

The authors declare that the research was conducted in the absence of any commercial or financial relationships that could be construed as a potential conflict of interest.
